# Albendazole and antibiotics synergize to deliver short-course anti-*Wolbachia* curative treatments in preclinical models of filariasis

**DOI:** 10.1073/pnas.1710845114

**Published:** 2017-10-23

**Authors:** Joseph D. Turner, Raman Sharma, Ghaith Al Jayoussi, Hayley E. Tyrer, Joanne Gamble, Laura Hayward, Richard S. Priestley, Emma A. Murphy, Jill Davies, David Waterhouse, Darren A. N. Cook, Rachel H. Clare, Andrew Cassidy, Andrew Steven, Kelly L. Johnston, John McCall, Louise Ford, Janet Hemingway, Stephen A. Ward, Mark J. Taylor

**Affiliations:** ^a^Research Centre for Drugs and Diagnostics, Department of Parasitology, Liverpool School of Tropical Medicine, Liverpool L3 5QA, United Kingdom;; ^b^TRS Laboratories, Athens, GA 30605

**Keywords:** filariasis, *Wolbachia*, macrofilaricide, albendazole, combination therapy

## Abstract

Elimination of filariasis requires a macrofilaricide treatment that can be delivered within a 7-day period. Here we have identified a synergy between the anthelmintic albendazole (ABZ) and drugs depleting the filarial endosymbiont *Wolbachia*, a proven macrofilaricide target, which reduces treatment from several weeks to 7 days in preclinical models. ABZ had negligible effects on *Wolbachia* but synergized with minocycline or rifampicin (RIF) to deplete symbionts, block embryogenesis, and stop microfilariae production. Greater than 99% *Wolbachia* depletion following 7-day combination of RIF+ABZ also led to accelerated macrofilaricidal activity. Thus, we provide preclinical proof-of-concept of treatment shortening using antibiotic+ABZ combinations to deliver anti-*Wolbachia* sterilizing and macrofilaricidal effects. Our data are of immediate public health importance as RIF+ABZ are registered drugs and thus immediately implementable to deliver a 1-wk macrofilaricide. They also suggest that novel, more potent anti-*Wolbachia* drugs under development may be capable of delivering further treatment shortening, to days rather than weeks, if combined with benzimidazoles.

## Significance

Filarial nematode infections, caused by *Wuchereria bancrofti*, *Brugia malayi* (elephantiasis), and *Onchocerca volvulus* (river blindness) infect 150 million of the world’s poorest populations and cause profound disability. Standard treatments require repetitive, long-term, mass drug administrations and have failed to interrupted transmission in certain sub-Saharan African regions. A drug cure using doxycycline, which targets the essential filarial endosymbiont *Wolbachia*, is clinically effective but programmatically challenging to implement due to long treatment durations and contraindications. Here we provide proof-of-concept of a radical improvement of targeting *Wolbachia* via identification of drug synergy between the anthelmintic albendazole and antibiotics. This synergy enables the shortening of treatment duration of macrofilaricidal anti-*Wolbachia* based treatments from 4 wk to 7 d with registered drugs ready for clinical testing.

Lymphatic filariasis (LF) and onchocerciasis present a significant global health burden, affecting an estimated 120 million and 38 million people, respectively (1–3). The causative agents of LF are the filarial nematodes *Wuchereria bancrofti*, *Brugia malayi*, and *Brugia timori*. Symptomatically, LF presents as lymphedema and hydrocele and, in more advanced cases, as elephantiasis (1, 2). Onchocerciasis is caused by the filaria *Onchocerca volvulus* and is associated with skin disease. In advanced untreated cases onchocerciasis can result in blindness caused by the immune response to migration and death of microfilariae (mf) in the eye (1, 3).

The standard antifilarial anthelmintics albendazole (ABZ), diethylcarbamazine (DEC), and ivermectin (IVM) are currently the mainstay of mass drug administration (MDA) elimination programs for filariasis. Standard antifilarial drug MDA regimens (delivered annually) do not deliver substantial curative efficacy but rather temporarily reduce the levels of mf in the blood or skin. While DEC and IVM are direct microfilaricides, ABZ inhibits mf production. ABZ/DEC combination treatment is used in MDA programs to eliminate LF, except in Africa, where an ABZ/IVM combination is used due to contraindications of DEC in onchocerciasis (1). Recently, a clinical trial administering a single triple-dose combination of IVM/DEC/ABZ has shown superior microfilaremia suppression in bancroftian filariasis for up to a year (4). These promising results have the potential to accelerate LF elimination goals, in situations where the absence of *Loa loa* and/or *O. volvulus* coendemicity precludes the risk of severe adverse events. For onchocerciasis elimination, MDA programs use IVM administered as a monotherapy in all endemic areas. Although targeting mf has proved effective in elimination of LF and onchocerciasis in certain country settings (5–7) the strategy has failed either to deliver expected outcomes or has not yet been deployed sustainably in many regions of sub-Saharan Africa (8–10). In particular, the use of IVM is problematic in geographical regions where *L. loa* is coendemic, as this can result in severe adverse reactions (SAEs) caused by drug-induced death and associated inflammation of blood-borne *L. loa* mf in the brain (11). These SAEs can result in encephalopathy, coma, and death (12). Epidemiological simulations also predict MDA will not interrupt transmission in certain scenarios (13, 14). Alternative treatment strategies are therefore required to achieve the WHO global elimination targets of LF and onchocerciasis. Ideally these should be short-course treatments delivering safe macrofilaricidal (curative) efficacy (15).

The bacterial symbiont *Wolbachia* is essential for the development, growth, and survival of many filarial parasites, including the causative agents of LF and onchocerciasis (15). Effective targeting of *Wolbachia* with tetracycline or rifamycin antibiotics induces growth retardation, embryostasis, and blockade of mf release in preclinical models of filariasis (16–18). Through a series of pivotal clinical trials, doxycycline (DOX) has demonstrated embryostatic, transmission-blocking, and curative efficacies against LF and onchocerciasis (19–24). Importantly, anti-*Wolbachia* treatments are safe to administer in loiasis coendemic areas because *L. loa* lacks *Wolbachia* symbiosis and DOX does not affect *Loa* microfilaraemias (22, 25). Further, the slow waning of mf in the circulation or the skin and gradual adult parasite death of the anti-*Wolbachia* mode of action avoids the inflammatory adverse reactions associated with rapid-acting direct filaricidal agents, in part by avoiding the liberation of *Wolbachia* as classical inflammatory inducers (21, 26–28). However, long treatment durations with DOX are required for significant antifilarial effects to be induced, related to a sustained >90% depletion level of the endosymbiont from filarial tissues after drug removal (29–31). Interestingly, sterilization of female filariae without significant macrofilaricidal activity (assessed up to 2 y after treatment) has been demonstrated clinically with a reduced treatment duration of DOX (3 wk vs. 4 wk), where *Wolbachia* was depleted >80% but <90% from nematode tissues (21). This suggests that a lower threshold depletion level of *Wolbachia* may still mediate sustained transmission-blocking activity in the treatment of LF and onchocerciasis (and disease-blocking activity in the case of onchocerciasis). Through pharmacokinetic–pharmacodynamic (PK-PD) modeling we have recently identified that the related second-generation tetracycline minocycline (MIN) may reduce overall treatment time in humans compared with DOX (18).

Rifampicin (RIF) has been shown to exhibit superior anti-*Wolbachia* potency in vitro and in vivo in models of LF and onchocerciasis compared with the tetracycline class (32–34). However, these observations have not translated into superior efficacy in clinical trials vs. DOX when administered at the “standard” 10 mg/kg dose for 2 or 4 wk to patients with onchocerciasis (35, 36). We have been able to explain this discrepancy based on RIF PKs and drug exposures recorded in preclinical models compared with humans. We have identified that a minimum dosage of RIF bioequivalent to 30–40 mg/kg in humans is required to deplete *Wolbachia* beyond the 90% threshold predictive of clinical cure (21, 29, 34). Reassuringly, fourfold dose elevations of RIF have recently been identified as safe when delivered for periods of 1 mo (37) in patients with TB, suggesting that RIF at a high dose could be deployed as a short-course macrofilaricidal drug for human use.

The global challenge is to develop a macrofilaricidal treatment that can be delivered in 7 d or less. In an effort to achieve these treatment times in this study we evaluated whether ABZ could enhance the anti-*Wolbachia* activities of the registered antibiotics MIN or RIF. Our results confirm substantial synergy between ABZ and both the tetracycline and rifamycin class of anti-*Wolbachia* drugs. Moreover, this synergy leads to long-term sterilizing effects and reduced treatment courses to 7 d. Unexpectedly, combining the most potent anti-*Wolbachia* regimen, high dose (HD)-RIF, with ABZ in a 7-d treatment also mediated an accelerated macrofilaricidal effect as well as significantly improving *Wolbachia* depletion beyond the 90% theshold in surviving adult female filariae, predictive of long-term asymbiotic macrofilaricidal activity.

## Results

### Benzimidazole Anthelmintics Do Not Directly Deplete Intracellular *Wolbachia*.

**Fig. 1.**
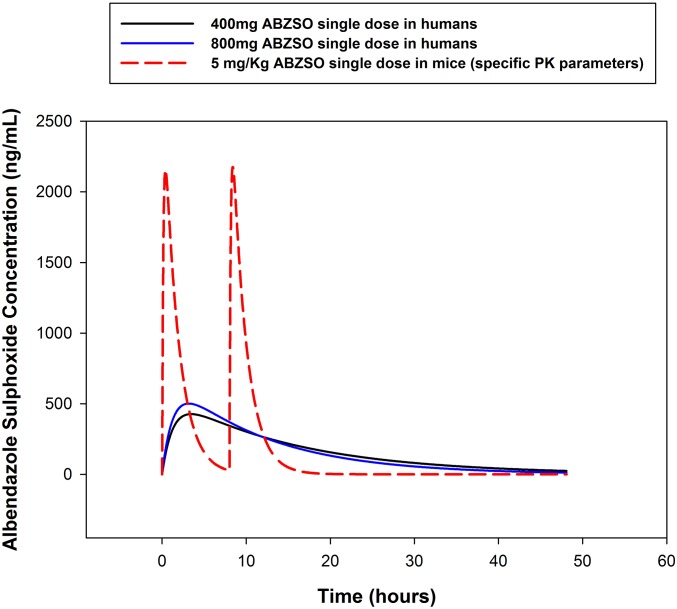
Human equivalent murine dosing regimens were calculated by Monte Carlo simulations.

The multiple-dose PKs of clinically bioequivalent ABZ were parameterized in CB.17 SCID mice (see *PK-PD Modeling*). ABZ is essentially a prodrug which is almost completely converted to the pharmacologically active metabolite albendazole sulphoxide (ABZ-SOX) in vivo; this is further converted to the pharmacologically inactive secondary metabolite albendazole sulphone (ABZ-SON). In our bioanalyses, the parental ABZ molecule was found to be below the limit of quantification in all sampled time points. Monte Carlo simulations were used to calculate murine dosage regimens that gave bioequivalent exposures of the active metabolite ABZ-SOX in mice to the standard clinical dose of 400 mg ABZ. The distribution of human exposures to ABZ were based on PK parameters from the literature (38–44). From simulations we determined a 5 mg/kg twice daily (*bid*) murine dosage regimen for ABZ closely emulated overall daily exposure of ABZ-SOX following a standard 400-mg clinical dose (mean exposure 400 mg ABZ in humans = 7.1 ± 5.0 μg⋅h/mL ABZ-SOX, and mean exposure 5 mg/kg *bid* in SCID mice = 7.7 ± 5.2 μg⋅h/mL ABZ-SOX; Fig. 1).

**Fig. 2.**
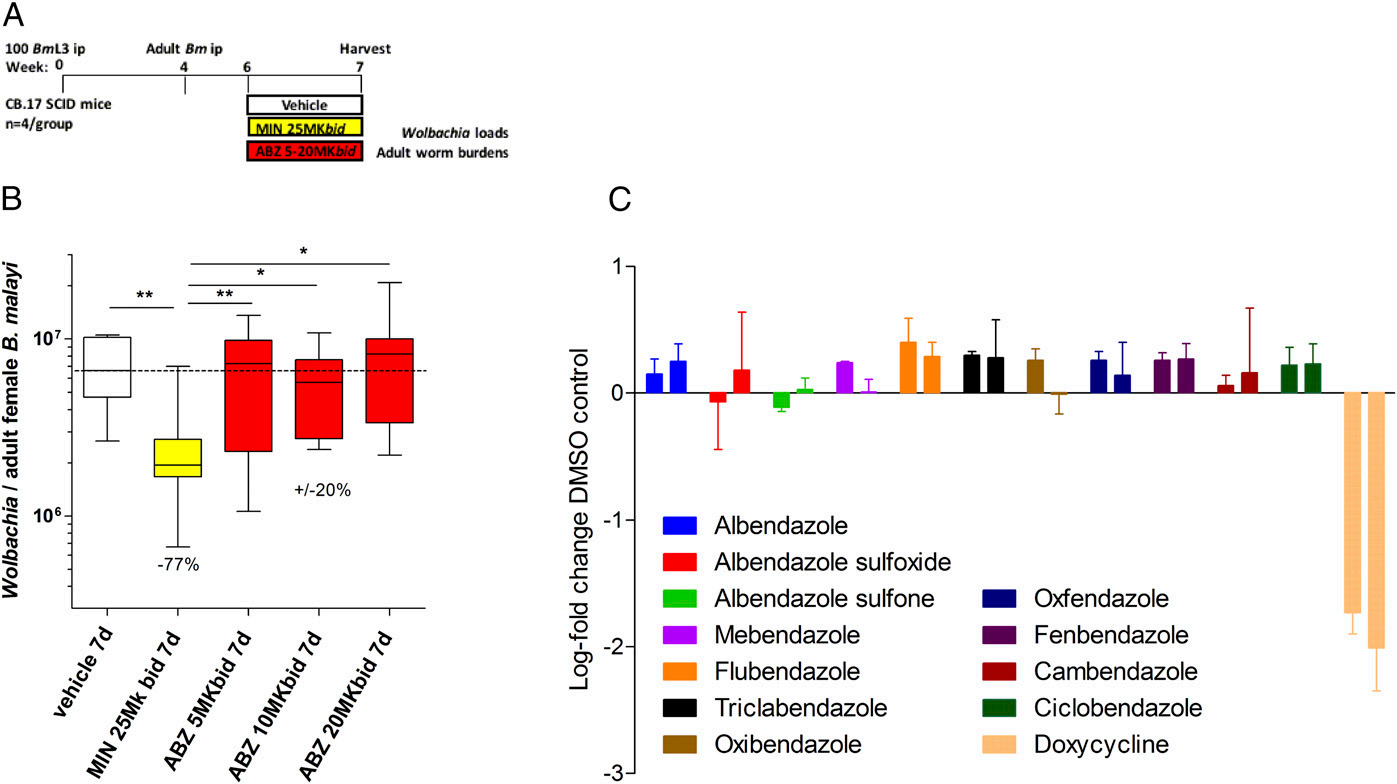
Schematic of drug treatment protocol (*A*). *Wolbachia* loads in *B. malayi* females immediately following 7-d exposure with MIN or ABZ at indicated doses (*B*). Box and whiskers are median, minimum/maximum *Wolbachia* surface protein (*wsp*) gene copy number, derived from a pool of 10 individual worms, sourced from groups of four SCID mice. Significant differences are assessed by Kruskal–Wallis one-way ANOVA (female worm *Wolbachia* depletions Kruskal–Wallis statistic: 21.79, *P* = 0.0002) with Dunn’s multiple tests: *P < 0.05, **P < 0.01. (*C*) Change in *Wolbachia* load compared with vehicle control following 7-d exposure to listed compounds (all 5 μM) within an *A. albopictus* cell line stably infected with *w*AlbB (C6/36 *w*AlbB). Bars are mean log-fold change (+SEM) from triplicate wells from two individual experiments.

Following the determination of the bioequivalent dose of ABZ in SCID mice we assessed whether pharmacologically relevant ABZ exposures had any direct effect on filarial *Wolbachia* in vivo. Groups of SCID mice with 6-wk-old adult *B. malayi* infections were dosed with vehicle control, bioequivalent 400 mg ABZ (5 mg/kg *bid*) or ABZ two- to fourfold in excess of bioequivalency (10 or 20 mg/kg *bid*) for +7 d. MIN (25 mg/kg *bid*) was used as an anti-*Wolbachia* positive control (Fig. 2*A*). One day after the last dose, total *Wolbachia* loads were enumerated from female *B. malayi* (Fig. 2*B*). Treatment with MIN for +7 d mediated a significant 77% median reduction in *Wolbachia* compared with vehicle controls. However, +7 d ABZ did not significantly reduce *Wolbachia* loads in *B. malayi* at the bioequivalent 400-mg human dose or up to fourfold higher exposures (Fig. 2*B*). Adult parasite loads were not affected by any ABZ treatment (*SI Appendix*, Table S1). We further assessed whether a range of benzimidazole (BZ) drugs, including the ABZ metabolites ABZ-SOX and ABZ-SON, could reduce *Wolbachia* titres in an in vitro insect cell assay at 5 μM continuous exposure over +7 d (Fig. 2*C*). No BZ compound was effective at depleting insect *Wolbachia*, whereas the DOX positive control reduced *Wolbachia* loads by between 1–2 log.

### ABZ Synergizes with Short-Course Antibiotic Treatments to Enhance Depletion of *Wolbachia* and Sustainably Block Filarial Embryogenesis.

**Fig. 3.**
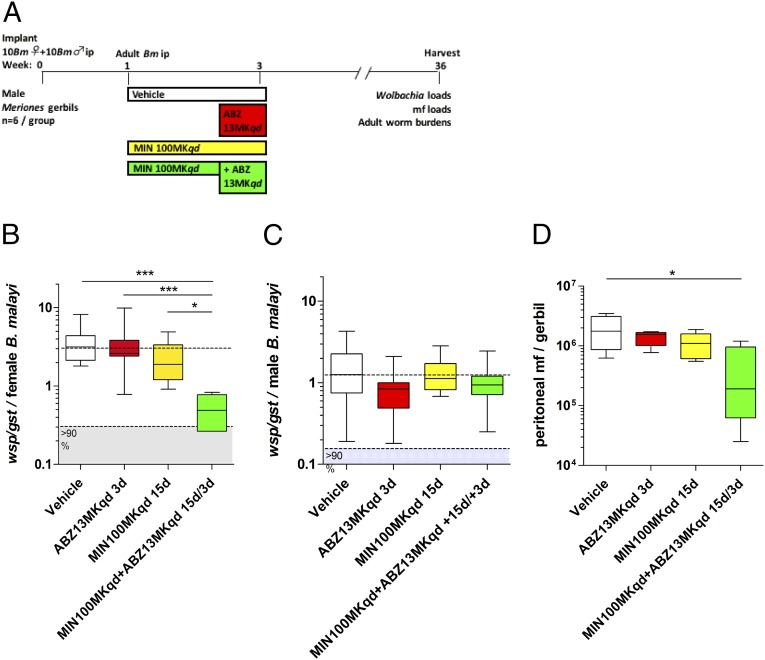
Schematic of drug treatment protocol (*A*). *Wolbachia* loads in *B. malayi* females (*B*), *Wolbachia* loads in *B. malayi* males (*C*), and peritoneal *B. malayi* microfilarial loads per gerbil (*D*), +8 mo postimplantation of adult filariae and commencement of 15-d vehicle, ABZ, MIN, or MIN+ABZ combination treatments (ABZ administered for final 3 d) at indicated doses. Box and whiskers are median, minimum/maximum *Wolbachia* surface protein (*wsp*) gene copy number, adjusted for heterogeneity in filarial age as a ratio to the *B. malayi GST* (*gst*) gene, derived from a pool of 10 individual worms, sourced from groups of six gerbils. Significant differences in *Wolbachia* load are assessed by Kruskal–Wallis one-way ANOVA, female worm *Wolbachia* depletions Kruskal–Wallis statistic: 21.2, *P* < 0.0001; male worm *Wolbachia* depletions Kruskal–Wallis statistic: not significant with Dunn’s multiple comparisons tests. *P < 0.05, ***P < 0.001. Significant differences in peritoneal mf number are assessed by one-way ANOVA with Holm–Sidak’s multiple comparisons tests *P < 0.05.

MIN is a superior anti-*Wolbachia* tetracycline in vivo compared with DOX yet is predicted to require a dosing period of >2 wk to deliver >90% depletion levels and blockade of embryogenesis in female filariae (18). Suboptimal dosages of tetracyclines lead to gradual *Wolbachia* recrudescence following drug removal (45). We therefore examined long-term effects of combining ABZ during the final 3 d of a suboptimal 15-d MIN regimen in a chronic gerbil model of brugian filariasis. Gerbils were implanted with 10 mature female and male *B. malayi* ip and after 1 wk were treated orally with MIN for 15 d [100 mg/kg once daily (*qd*)], ABZ (13 mg/kg *qd*) or MIN for 15 d in combination with ABZ for the final 3 d of dosing (Fig. 3*A*). Gerbils were left for a protracted washout period of 8 mo following the start of treatment before adult filariae and mf were recovered and enumerated. qPCR analysis confirmed that neither ABZ nor MIN monotherapy mediated a significant *Wolbachia* depletion within female *B. malayi*, compared with vehicle control worms (ABZ: 17% median depletion vs. vehicle, MIN: 40% median depletion). In comparison, MIN+ABZ combination significantly synergized in the depletion of *Wolbachia* from adult female *B. malayi* by approximately twofold (MIN+ABZ: 84% *Wolbachia* depletion). The synergy in *Wolbachia* depletion was specific to female *B. malayi* because no drug treatment significantly impacted *Wolbachia* loads within male *B. malayi* after +8 mo washout (Fig. 3*C*). The parasitological effects of targeting *Wolbachia* with ABZ, MIN, or MIN+ABZ combination were assessed. All vehicle and drug-treated animals contained viable adult filariae +8 mo after implantation and no significant differences were apparent in adult yields (*SI Appendix*, Table S2). Vehicle control-treated animals contained 1.9 × 10^6^ ± 0.5 × 10^6^ (mean ± SEM) viable peritoneal mf (Fig. 3*D*). Gerbils treated with MIN or ABZ monotherapies contained similar levels of mf, whereas the MIN+ABZ combination treatment had significantly reduced peritoneal mf reduced on average by 77.9% (0.4 × 10^6^ ± 0.2 × 10^6^ viable peritoneal mf).

**Fig. 4.**
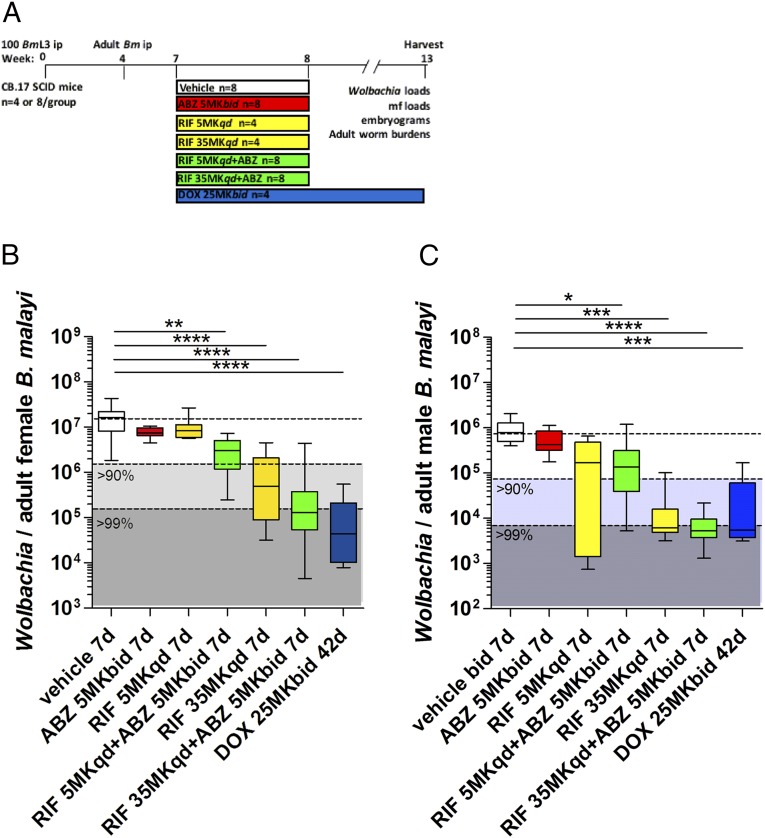
Schematic of drug treatment protocol (*A*). *Wolbachia* loads in *B. malayi* females (*B*) or males (*C*), +13 wk postinfection and +6 wk after commencement of 7-d vehicle, ABZ, RIF, ABZ+RIF combination treatments, or DOX treatment for +42 d at indicated doses. Box and whiskers are median, minimum, and maximum *Wolbachia* surface protein gene copy number, derived from a pool of 10 individual worms, sourced from groups of three to four mice. Significant differences are assessed by Kruskal–Wallis one-way ANOVA (female worm *Wolbachia* depletions Kruskal–Wallis statistic: 49.7, *P* < 0.0001; male worm *Wolbachia* depletions Kruskal–Wallis statistic: 37.1, *P* < 0.0001) with Dunn’s multiple tests, indicated as *P < 0.05, **P < 0.01, ***P < 0.001, or ****P < 0.0001 (only tests compared with vehicle are shown).

Because of the synergism observed at the level of *Wolbachia* depletion and reduced mf burdens following MIN+ABZ combination, we next evaluated whether synergy was also observable when combining ABZ with the more potent anti-wolbachial agent RIF. RIF regimens, previously defined as bioequivalent to either “standard” (∼10 mg/kg) or “high” (∼30 mg/kg) clinical regimens (34) were tested. Groups of SCID mice with 7-wk-old adult *B. malayi* infections were dosed with vehicle control, bioequivalent 400 mg ABZ (5 mg/kg *bid*) bio-equivalent, standard-dose (SD)-RIF (5 mg/kg *qd*), HD-RIF (35 mg/kg *qd*) or combinations of SD-RIF or HD-RIF+ABZ, all for +7 d (Fig. 4*A*). An additional group of SCID mice were dosed with 100 mg bioequivalent DOX (25 mg/kg *bid* DOX) for +42 d, as a positive control (46). Six weeks after dose commencement, total *Wolbachia* loads were enumerated from female and male *B. malayi* (Fig. 4 *B* and *C*). As previously defined, +7 d bioequivalent ABZ did not significantly impact *Wolbachia* loads in female *B. malayi* and, further, did not significantly reduce *Wolbachia* within male *B. malayi*. SD-RIF also did not significantly reduce *Wolbachia* load in female *B. malayi*. Combinations of SD-RIF+ABZ enhanced the *Wolbachia* depletion in female *B. malayi* approximately twofold compared with SD-RIF alone, which was significant compared with vehicle control levels (48.5% depletion, SD-RIF monotherapy vs. 83.3% median depletion SD-RIF+ABZ). In contrast, *Wolbachia* depletion in male *B. malayi* was not enhanced following treatment with the SD-RIF+ABZ combination (83% vs. 79%). As demonstrated in previous studies (34), HD-RIF monotherapy induced significant, >90% threshold levels of *Wolbachia* depletion in female *B. malayi* (97.3%) and >99% threshold depletion in male *B. malayi* (99.2%). However, combinations of HD-RIF+ABZ elevated the median *Wolbachia* depletion level further from >90% to >99% threshold efficacy compared with HD-RIF monotherapy (99.3% vs. 97%). This anti-*Wolbachia* synergy was again specific to female filariae, because the degree of *Wolbachia* depletion in male *B. malayi* following HD-RIF+ABZ compared with HD-RIF monotherapy was unchanged (99.3% vs. 99.2%). The level of *Wolbachia* depletion in female or male *B. malayi* following a 42-d, 100-mg human dose equivalent of DOX (25 Mk *bid*) is given as a comparison where a 99.7% median depletion was evident in female *B. malayi*; only the HD-RIF+ABZ combination achieved a similar, >99% threshold level of depletion matching long-course DOX, in female *B. malayi*, when dosed for +7 d.

**Fig. 5.**
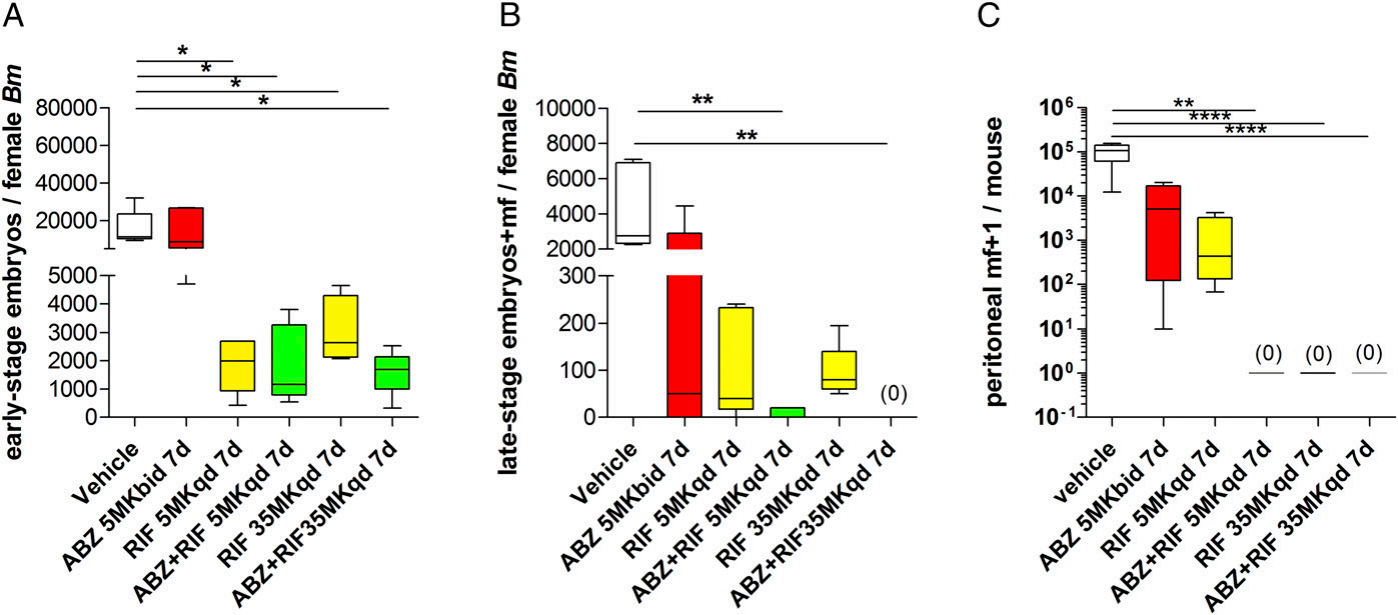
Quantification of early intrauterine embryonic stages (*A*) and late intrauterine embryonic stages (*B*) in *B. malayi* females and accumulations of mature, motile i.p. *B. malayi* mf (*C*), +13 wk postinfection and +6 wk after commencement of 7-d vehicle, ABZ, RIF, or combination treatments at indicated doses. Box and whiskers are median levels, minimum/maximum of indicated uterine stage/s or mature, motile i.p. mf. Embryogram data are derived from a pool of five individual females, sourced from groups of three to four mice; i.p. mf counts are derived from four mice per group (RIF monotherapies), six mice per group (ABZ monotherapy), or eight mice per group (vehicle and RIF+ABZ combinations). Significant differences are assessed by one-way ANOVA with Holm–Sidak’s multiple comparison tests (early-stage embyos/female worm *F* = 6.537, *P* = 0.0006) or Kruskal–Wallis one-way ANOVA with Dunn’s multiple comparisons tests (late-stage embryos/female worm Kruskal–Wallis statistic: 19.8, *P* = 0.0014, peritoneal mf/mouse Kruskal–Wallis statistic: 35.5, *P* < 0.0001). Significance of multiple comparisons tests are indicated: *P < 0.05, **P < 0.01, or ****P < 0.0001.

The parasitological consequences of RIF+ABZ anti-*Wolbachia* synergy within female *B. malayi* were scrutinized. Analysis of embryogenesis in female *B. malayi* following ABZ, RIF, or RIF+ABZ combination treatments was undertaken (Fig. 5). Early-stage embryos were not significantly reduced in female *B. malayi* uteri following standard-dose ABZ 7-d monotherapy vs. vehicle controls (Fig. 5*A*). Further, late-stage embryonic stages (“pretzel” stage, coiled embryos and stretched intrauterine mf) were variable and not significantly different following ABZ monotherapy vs. vehicle controls (Fig. 5*B*). Short-course RIF treatments, irrespective of dose and combination with ABZ, significantly reduced early-stage embryos vs. either vehicle controls or ABZ monotherapy treatment (Fig. 5*A*). However, only combinations of SD-RIF+ABZ or HD-RIF+ABZ mediated significant reductions in late-stage intrauterine developmental stages vs. vehicle controls (Fig. 5*B*). In the case of HD-RIF+ABZ, a complete absence of late-stage embryonic stages was apparent within female *B. malayi*. When measuring fecundity, accumulations of mature, motile mf were consistently present in vehicle controls (eight of eight mice, 0.11 × 10^6^ median viable peritoneal mf, range 0.06–0.14 × 10^6^). ABZ (six of six mice) or standard-dose RIF (four of four mice) groups also contained viable mf and peritoneal microfilarial loads were not significantly reduced vs. vehicle group (Fig. 5*C*). In comparison, mature mf were completely absent in the peritonea of mice treated with HD-RIF monotherapy (zero of four mice) or combinations of either SD-RIF+ABZ (zero of eight mice) or HD-RIF+ABZ (zero of eight mice).

**Fig. 6.**
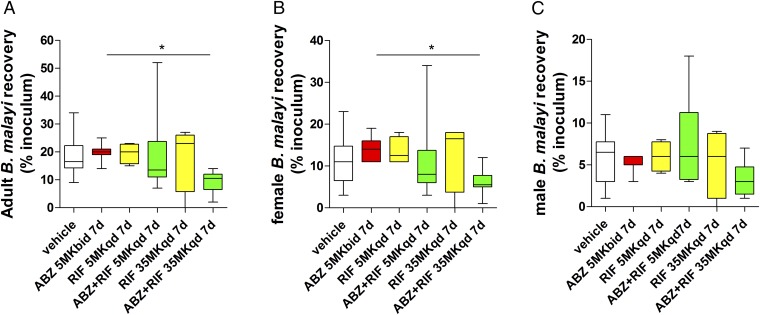
Quantification of total (*A*), female (*B*), and male (*C*) *B. malayi* worm burdens +13 wk postinfection and +6 wk after commencement of 7-d vehicle, ABZ, RIF, or combination treatments at indicated doses. Box and whiskers are median percent recoveries (100 *Bm*L3 infections) and minimum/maximum of mature, motile adult stages from four mice per group (RIF monotherapies) seven mice per group (ABZ monotherapy) or eight mice per group (vehicle and RIF+ABZ combinations). Significant differences are assessed by Kruskal–Wallis one-way ANOVA with Dunn’s multiple comparisons tests (total worm burden Kruskal–Wallis statistic: 12.6, *P* = 0.0274; female worm Kruskal–Wallis statistic: 11.8, *P* = 0.0379). Significance of multiple comparisons tests are indicated as *P < 0.05.

Total *B. malayi* adult worm burdens were significantly reduced following treatment with HD-RIF+ABZ vs. ABZ monotherapy (47.5% median reduction) (Fig. 6). No other treatment significantly affected total worm burdens recovered 6 wk postdosing. When examining sex-specific effects, female worm recoveries were significantly reduced following HD-RIF+ABZ vs. ABZ monotherapy (60.7% median reduction) while male worm burdens were not significantly different in any treatment group.

### Lack of Evidence for a Drug–Drug Interaction When Coadministering ABZ+RIF.

**Fig. 7.**
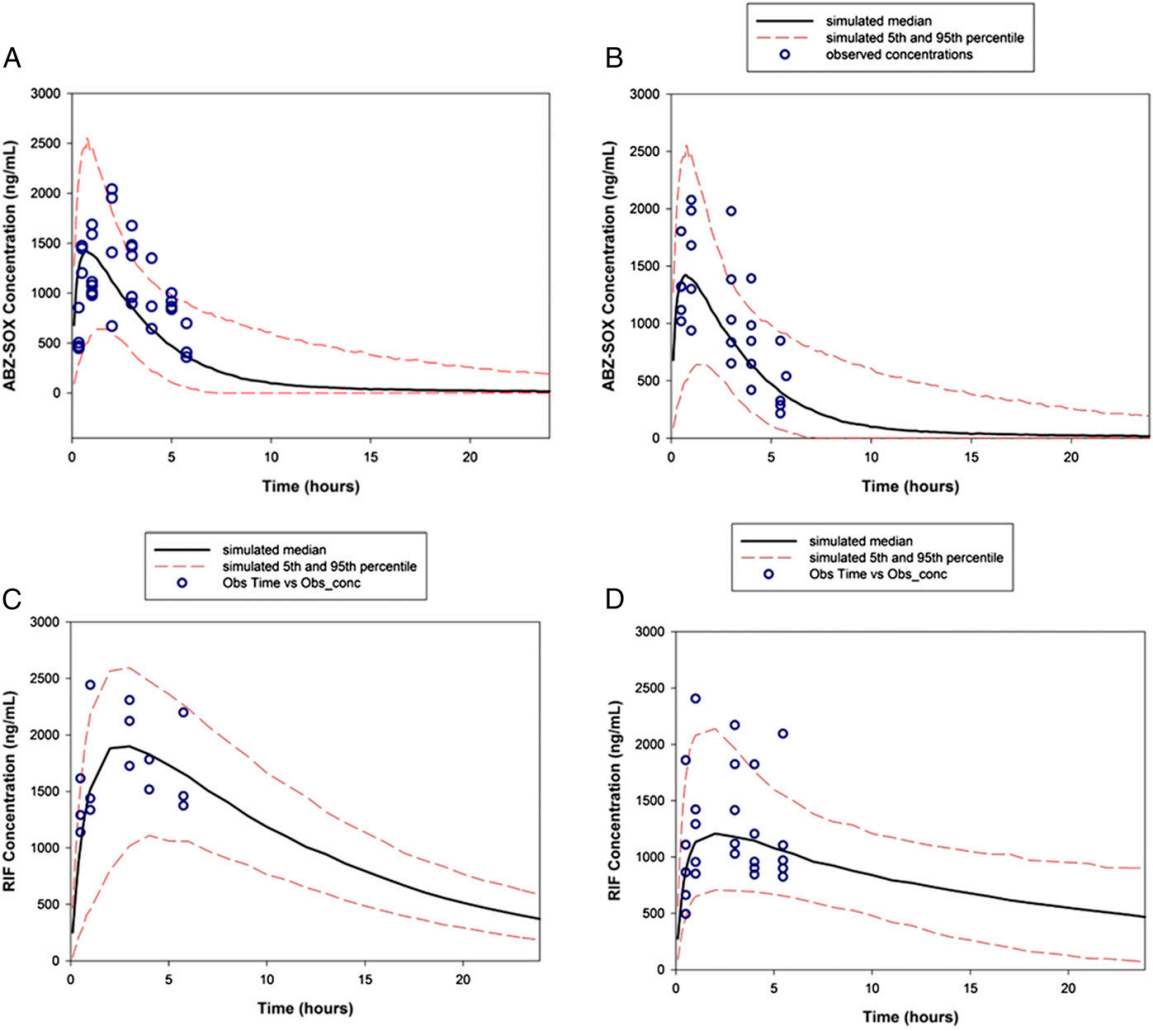
Simulated PK profiles (day 1) for (*A*) 5-d 5 mg/kg *bid* ABZ regimen. (*B*) ABZ in ABZ+RIF combination 5-d regimen. (*C*) The 5-d 5 mg/kg *qd* RIF regimen (day 1). (*D*) RIF in ABZ+RIF combination regimen. For the simulated PK profiles the solid line represents the median profile and the red dashed lines represent the 5th and 95th percentile PK profiles and blue circles experimentally sampled concentrations.

RIF is a known autoinducer of the cytochrome P450 system, although we have shown previously that there is no autoinduction after a 7-d dose of RIF in our mouse model (34). To scrutinize whether coadministration of RIF+ABZ altered the systemic exposures of either RIF or the ABZ active metabolite ABZ-SOX, the PK of RIF and ABZ-SOX after combination treatment for +5 d at clinically bioequivalent dosages was characterized. For RIF, a standard 600-mg dose in humans yields exposures of 55.1 μg⋅h/mL (34), whereas in mice a *qd* 5 mg/kg dose administered gives an exposure 46.7 ± 16.0 μg⋅h/mL A one-compartment model with absorptive gut compartment was found to describe the data best for both monotherapy and combination therapy data. Fig. 7 shows the simulated profiles for clinically bioequivalent ABZ and RIF when given separately and in combination with experimentally sampled drug concentrations superimposed. By comparing the PK parameters of clinically bioequivalent dosages of RIF and ABZ-SOX we determined that the chronic exposures for both ABZ-SOX and RIF did not significantly vary when given in combination in comparison with those determined for the monotherapies (Table 1). This result was confirmed using sparsely sampled concentrations from monotherapy and combination therapy efficacy studies, as these were found to be routinely within the simulated bounds for the monotherapy PK profiles and there was no systematic deviation for combination therapy drug concentrations in comparison with the monotherapy concentrations (*SI Appendix*, Fig. S4). Levels of the terminal ABZ-SON metabolite were similarly unaffected by combination chronic dosing with RIF (*SI Appendix*, Fig. S4 *C* and *D*).

**Table 1.** Pharmacokinetic parameters for chronically dosed clinically bioequivalent RIF, ABZ, and RIF+ABZ

Parameter	ABZ monotherapy (*n* = 8)	ABZ combination therapy (*n* = 5)	RIF monotherapy (*n* = 3)	RIF combination therapy (*n* = 5)
CL/F, mL/(h⋅kg)	868 ± 323	724 ± 256	167 ± 4	156 ± 80
V/F, mL/kg	2,187 ± 846	2,572 ± 694	1,871 ± 314	3,857 ± 1,167
*k*_*a*_	4.2	7.8	1.0 ± 0.5	2.4 ± 0.7
AUC_24__h—day__5_, µg⋅h/mL	7.7 ± 5.2	8.9 ± 4.2	46.7 ± 16.0	30.0 ± 11.5

AUC_24 h—day 5_, 24-hour exposure for day 5; CL/F, apparent clearance; *k_a_*, rate constant for absorption; V/F, apparent volume of distribution.

## Discussion

Our data reveal a pharmacological drug synergy between registered classes of antibiotics when combined with the standard antifilarial drug ABZ in targeting the filarial symbiont *Wolbachia*. The impact of this synergy is to reduce treatment time to as little as 1 wk to substantially deplete symbionts with concomitant long-term embryostatic activities, total inhibition of mf production, and accelerated killing of female worms.

*Wolbachia* populate two major tissues within female filariae, the hypodermal chord syncytium and the germline (47). The latter population is thought to become stably infected via transfer of hypodermal *Wolbachia* into germline stem cells during development of female gonads within fourth-stage larvae, at 8–11 d postinfection (48). *Wolbachia* then rapidly divide during oogenesis (35–50 bacteria per egg) (49). Following fertilization of female worms, *Wolbachia* further divide and spread in zygotes, by asymmetric mitotic segregation, into the embryonic stem cell precursor of the lateral chord and subsequently expand within the primordial microfilarial hypodermal chord (100–200 *Wolbachia* per mf; refs. 48–50). At this part of the life cycle, *Wolbachia* symbiosis provides an essential source of nucleotides and micronutrient pathways (FAD, heme, and riboflavin) to support the high demand on biosynthesis during rapid oogenic/embryonic cell division (47). Thus, effective depletion of the germline *Wolbachia* filarial population may lead to a highly localized deficiency in growth and survival factors necessary for embryogenesis. Indeed, antibiotic treatment of *B. malayi* and effective depletion of *Wolbachia* induces apoptosis of embryonic and uterine tissues, which is consistent with permanent sterility (51).

Because anti-*Wolbachia* synergy was only reproducibly observed in female rather than in male adult filarial tissues following ABZ+antibiotic drug treatments, we hypothesize that the synergistic activity of ABZ+antibiotic combination targets the germline *Wolbachia* population, preventing their division, spread, and appropriate asymmetric localization in oocytes and embryos. While the precise mechanism by which ABZ augments anti-*Wolbachia* activity remains to be identified, as a nematode β-tubulin polymerization inhibitor ABZ potentially interferes with intracellular locomotion of dividing *Wolbachia* along microtubules, thus enhancing a bacteriostatic antibiotic mode of action. Albeit using concentrations far in excess of the in vivo pharmacological range, *Wolbachia* division along microtubules within a *Drosophila* cell line or *B. malayi* can be disrupted following in vitro ABZ exposures, causing a phenomenon of *Wolbachia* elongation (52). The effect of ABZ on *Wolbachia* elongation was limited to the germline population in female *B. malayi*. Further, because BZs target rapidly dividing cells and have established embryotoxic effects (53, 54), temporary disruption of nematode oogenesis and embryogenesis by ABZ may prevent residual *Wolbachia* titres from recrudescing within the germline niche following removal of otherwise suboptimal, short-course antibiotic exposures.

We adopted PK modeling to align our preclinical registered drug exposures with documented human exposures. Thus, we are encouraged that the synergy observed in our SCID mouse preclinical model of filariasis is likely to bridge into the clinical setting, using combinations of safe dosages for which there is already considerable human experience. Importantly, this may allow for treatment shortening of 7 d or less with an anti-*Wolbachia* targeted approach, to exert permanent sterility of LF and *Onchocerca* infections. Because both ABZ and RIF are safe to administer in loiasis patients, this potentially could translate to a one-off short-term administration strategy delivering sterilizing and curative activities against LF and onchocerciasis in patients coinfected with *L. loa* infection, in hard-to-reach areas of forested central and west Africa. These observations come at an important time as the related BZ drug flubendazole, which is more potent than ABZ in preclinical models and demonstrably macrofilaricidal in onchocerciasis patients when administered as an intramuscular injection, is under development as an oral reformulation (55) capable of delivering a macrofilaricide effect. However, rigorous regulatory preclinical safety testing has confirmed that this drug is too toxic for human use. This observation may blight the developability of other directly acting BZs such as the veterinary BZ oxfendazole, which is undergoing repurposing in humans as a neglected tropical disease indication with the potential for onchocerciasis testing in the future (56). Therefore, if synergy between anti-*Wolbachia* drugs and the BZ class is consistently demonstrable in preclinical models, there is the potential to explore a strategy of combining antibiotics with lowered doses of novel human BZ indications with more robust safety profiles, to exert permanent sterility and cure of filarial infections with single administration period of 7 d or less.

Recently, ABZ monotherapy given semiannually has been demonstrated to deliver significant long-term (2 y) reductions in circulating mf in African LF patients (57). Trials in onchocerciasis with variable length and dose of ABZ (3–10 d) have provided evidence that, as a monotherapy, ABZ can mediate a degree of filarial embryotoxicity and impact skin microfilarial levels responsible for disease and transmission (58, 59). Further, in a recent pilot trial combining ABZ for the final 3 d of a 3-wk DOX treatment in onchocerciasis patients demonstrated increased efficacy at the level of embryogenesis blockade compared with DOX monotherapy, demonstrating that ABZ+antibiotic synergy translates to the clinic (60). Our preclinical data provide strong proof-of-concept that by combining more potent anti-*Wolbachia* regimens with ABZ a total block of embryogenesis can be mediated within a 7-d timeframe. These observations provide a strong rational for immediate clinical evaluation of these synergistic combinations in LF and onchocerciasis patients. Future work should also examine the effects of combining the “next-generation” anti-*Wolbachia* candidates being developed from drug discovery programs (61, 62), which have improved potency and reduced treatment timeframes compared with DOX, with ABZ (or other repurposed/reformulated BZ anthelmintics) with the goal of reducing treatment timeframes to less than 7 d.

The potential translational medicine impact of our documented antifilarial drug combination synergy is a treatment shortening to days, rather than weeks, of a safe, well-tolerated macrofilaricide regimen, compatible with all target populations, with the potential to deliver “end-game” elimination of LF and onchocerciasis.

## Materials and Methods

### Parasites.

The *B. malayi* (*Bm*) filarial nematode parasite life cycle [TRS strain (63)] was maintained in mosquitoes and susceptible *M. unguiculatus* gerbils at Liverpool School of Tropical Medicine (LSTM) or TRS laboratories. Infective *Bm* larvae (*Bm*L3) were bred via procedures as previously described (64). Briefly, mf collected from infected gerbils by catheterization (65) were fed to female adult *Aedes aegypti*; mf were mixed with human blood and fed to mosquitoes using an artificial membrane feeder (Hemotek). *Bm*L3-stage larvae were propagated by rearing the blood-fed mosquitoes for 14 d. *Bm*L3 larvae were then harvested from infected mosquitoes by crushing and purification (64).

### Animals.

Male BALB/c CB17 SCID mice were obtained from Harlan Laboratories. Outbred *M. unguiculatus* gerbils were bred at LSTM or TRS laboratories. All animals were housed under specific pathogen-free conditions at approved animal housing facilities. Ethical approval was obtained for all animal experiments at LSTM from the relevant animal welfare committees at the University of Liverpool and LSTM. Experiments were conducted according to Home Office or US national requirements.

### Parasite Infections and Implantations.

One hundred freshly isolated *B. malayi* L3 larvae were injected intraperitoneally into CB.17 SCID mice. Inoculation efficiencies were confirmed by needle washout. For *B. malayi* implantations, gerbils were anesthetized and 10 mature female and male *B. malayi* were surgically implanted into the peritoneum, as previously described (64).

### Preparation and Administration of Drug Compounds.

All drugs were given at stated concentrations and durations, as a solution via oral gavage, 6–7 wk after infection or 1 wk after implantation. Rodents were administered with 100 µL of ABZ, DOX, MIN, or RIF. For combination therapy, 200 µL containing a 1:1 mixture of two compounds was administered. DOX and MIN were dissolved in water. RIF was dissolved in 55% polyethylene glycol 300, 25% propylene glycol, and 20% water and ABZ was dissolved in standard suspension vehicle composed of 0.5% carboxymethyl cellulose, 0.5% benzyl alcohol, 0.4% Tween 80, and 0.9% NaCl. All reagents were purchased from Sigma unless otherwise stated.

### Parasitological Readouts.

Mice were necropsied at either 7 or 13 wk postinfection as indicated. RPMI medium was used in peritoneal washes to recover adults and mf. After isolation, adult worms were stored overnight in fresh medium at 4 °C before being washed in cold PBS. Adult stages were then enumerated and their sex determined. For qPCR analysis of *Wolbachia* loads, 10–15 female adults were analyzed per treatment group, derived from each screen. Between two and four female worms were selected, randomly, from individual mice, to avoid bias due to intragroup dosing variation. mf were pelleted from peritoneal washings by centrifugation (300 g, 5 min, 4 °C, low brake) and resuspended in 1–2 mL of medium and the total mf quantity contained within peritoneal lavage was enumerated by microscopy.

### Quantification of *Wolbachia* Bacteria Numbers Using qPCR Assay.

DNA extraction from individual female worms was performed using a DNeasy Blood and Tissue Kit (Qiagen) in accordance with the manufacturer’s instructions. Quantification of *Bm Wolbachia wsp* copy numbers was performed using qPCR as previously described (50).

### Determination of in Vitro Potencies.

The anti-*Wolbachia* potency of a range of BZs was determined in vitro, utilizing an insect cell screening assay as described previously (61). Briefly, the mosquito (*Aedes albopictus*) derived cell line (C6/36), stably infected with *Wolbachia pipientis* (*w*AlbB) (C6/36 *w*AlbB) were incubated with a panel of BZ compounds, DOX (all 5 μM, dissolved in DMSO), or 1% DMSO control for 7 d with 2,000 cells per well on a 384-well plate (CellCarrier-384 Ultra; PerkinElmer) in Leibovitz media (Life Technologies) supplemented with 20% FBS (Fisher Scientific), 2% tryptose phosphate broth (Sigma-Aldrich), and 1% nonessential amino acids (Sigma-Aldrich). The end-point readout utilized DNA staining of both the host cell nuclei and intracellular *Wolbachia* (SYTO11) combined with a high-content imaging system (Operetta; PerkinElmer) and analyzed using the associated Harmony software through a cytoplasm texture analysis.

### Pharmacokinetic Studies in CB.17 SCID Mice.

Rich PK studies were conducted to determine the PKs of ABZ 5 mg/kg *bid* and RIF 5 mg/kg *qd* regimens administered orally as monotherapies for 5 d. PK of a single dose of 10 mg/kg in uninfected BALB/c CB.17 SCID mice was also determined. ABZ, the primary metabolite ABZ-SOX, and the secondary metabolite ABZ-SON were all quantified in these rich PK studies. These studies were conducted to assess PK at steady state and also linearity as bioavailability-limited dose-dependent PKs have been reported in humans (41, 42).

The PKs of RIF and ABZ when given in combination were also characterized via rich PK studies in uninfected CB.17 SCID mice (weight 24–28 g); RIF and ABZ were administered in combination at 5 mg/kg *qd* and 5 mg/kg *bid* for 5 d. All dosage regimens were administered via oral gavage in the appropriate vehicles, matching administrations used in PD studies. Blood samples were collected using microsampling from the tail vein for all studies. For the monotherapy studies serial blood samples were collected up to 24 h after a single dose and after the last dose for the chronic dosing regimen. For the combination therapy study serial blood samples were collected up to 6 h after dosing.

In all cases 20-µL blood samples were collected using a pipette with a preheparinized tip. Samples were immediately lysed with 40 µL of ice-cold double-distilled (dd) H_2_O and then frozen at −80 °C until time of analysis. Sparse samples were obtained from the PD studies in the *B. malayi* CB.17 SCID murine infection model to ensure that drug exposures after combination therapies were in line with those observed in the rich PK studies.

### Bioanalysis.

RIF and ABZ whole-blood drug concentrations were quantified using liquid chromatography–MS on a UPLC (ultrahigh-pressure liquid chromatography) system linked to a triple-quadruple TSQ Quantum Access mass spectrometer (Thermo Scientific) with a heated-electrospray ionization source.

For RIF chromatic separation was achieved using previously validated protocols (34). For ABZ and its metabolites ABZ-SOX and ABZ-SON chromatographic separation was achieved using an isocratic method consisting of methanol:water (50:50, vol/vol). Separation was achieved using reverse-phase chromatography carried out on a Hypersil C8 Gold column (50 × 2.1 mm, 1.9-μm particle size; Thermo Scientific). All methods used the appropriate internal standards and were validated using Food and Drug Administration guidelines which are internationally recognized (66).

To a 20-μL aliquot of blood lysate 180 μL of ddH_2_O was added plus 800 μL of dichloromethane/hexane/methyl-*tert*-butyl ether (1/1/1) containing 1,000 ng/mL of internal standard sulfisoxazole. Samples were then vortexed for 10 s and centrifuged at 13,000 × *g* for 10 min. A 750-μL aliquot was then taken from the supernatant and evaporated to dryness at 30 °C, under a gentle stream of nitrogen. The residue was then reconstituted in 120 μL of methanol:water (50:50, vol/vol) and transferred to 300-μL clean, dry, glass vials. A 10-μL aliquot was then subjected to UPLC-MS/MS analysis.

For RIF, protocols from previous studies for calibration and quantification were followed (34). For ABZ, ABZ-SOX, and ABZ-SON, a whole-blood calibration curve (range 50–2,000 ng/mL) was run alongside blood samples; quality-control samples at low 50 ng/mL, medium 1,000 ng/mL, and high 1,500 ng/mL concentrations were used for ABZ, ABZ-SOX and ABZ-SON, respectively. All standard curves were described using an equal weighted linear regression equation using the data acquisition software LC Quan (version 2.5.6; Thermo Scientific). The correlation coefficients (r^2^) for ABZ, ABZ-SOX, and ABZ-SON calibration curves all exceeded 0.97.

The lower limit of quantification (LLOQ) is described as the lowest possible concentration on the calibration curve and is validated using an LLOQ sample which has an accuracy determined by its relative error ± 20% and a precision determined by its relative SD of less than 20%.

### PK-PD Modeling.

All pharmacokinetic modeling and simulations was performed using Pmetrics (67) within R version 3.1.0 (68). PK models were built for both monotherapies and combination therapies using sample concentrations from rich PK studies. ABZ is almost completely converted to the active metabolite ABZ-SOX in-vivo, then it is further converted to the pharmacological inactive secondary metabolite ABZ-SON. Given the dose dependency reported in previous PK studies in clinical and murine studies, dose-specific parameters were fitted. Pharmacokinetic models fitting drug concentrations for the pharmacologically active metabolite of interest ABZ-SOX were constructed. The final model incorporated a one-compartment model with an absorptive gut compartment for oral dosing as detailed by the differential equations **1a** and **1b** (*SI Appendix*, Fig. S1):

$$\frac{dX_{1}}{dt}=-k_{a}X_{1}$$ [1a]

$$\frac{dX_{2}}{dt}=k_{a}X_{1}-\left(\frac{CL}{V}\right)X_{2},$$ [1b]

where *X*_1_ and *X*_2_ are the amounts of ABZ and ABZ-SOX in the absorptive and central compartments, representing gut and systemic circulation, respectively. The pharmacokinetic parameters *k*_*a*_, *CL*, and *V* denote the absorption rate constant, apparent clearance, and volume of distribution, respectively. In this instance *k*_*a*_ is a lumped parameter including rate of conversion from ABZ to ABZ-SOX as the parent drug was found to be below the limit of quantification in all sampled time points for monotherapy and combination therapy. A two-compartment model with absorptive gut compartment simultaneously fitting ABZ-SOX and ABZ-SON was also constructed (*SI Appendix*, Figs. S2 and S3 and Table S3). The final model showed a good fit of both metabolites but was not used further as the primary metabolite model was a slightly better fit for the pharmacologically active metabolite and PK parameters were simpler to interpret.

Model fitting was performed using protocols defined previously (69, 70). Briefly, the goodness-of-fit of the observed/predicted values (population and individual predictions) were assessed by linear regression (intercept close to 0, slope close to 1), the coefficient of determination of the linear regression (R^2^ close to 1.0), and log-likelihood values. A statistically significant improvement in the log-likelihood value (*P*, 0.05) was required for a more complex model to be supported.

### Statistical Tests.

The continuous variables *wsp* copy number, total *B. malayi* worm count, female *B. malayi* worm count, and male *B. malayi* worm count were not normally distributed following log transformations, determined by D’Agostino and Pearson omnibus tests. Therefore, statistical significance was assessed by Kruskal–Wallis tests followed by Dunn’s multiple comparisons tests post hoc comparing three or more groups.

The continuous variable total peritoneal mf count was normally distributed, determined by D’Agostino and Pearson omnibus tests. Statistical significance was assessed two-tailed, one-way ANOVA with Holm–Sidak’s multiple comparisons tests post hoc comparing three or more groups. Significance level was set to alpha = 0.05.

## Supplementary Material

Supplementary File
