# Supplementary material for: Albendazole and antibiotics synergize to deliver short-course anti-Wolbachia curative treatments in preclinical models of filariasis
Source: Proc Natl Acad Sci U S A. 2017 Oct 23;114(45):E9712–21. doi: 10.1073/pnas.1710845114 (PMC5692564; doi:10.1073/pnas.1710845114)
Supplement: Supplementary File [file pnas.1710845114.sapp.pdf]

Supporting Information

Albendazole and antibiotics synergize to deliver short-course anti-*Wolbachia* curative treatments in preclinical models of filariasis

Joseph D. Turner[1*], Raman Sharma[1*], Ghaith Al Jayoussi[1], Hayley E. Tyrer[1], Joanne Gamble[1], Laura Hayward[1], Richard Priestly[1], Emma A. Murphy[1], Jill Davies[1], David Waterhouse[1], Darren A. N. Cook[1], Rachel H. Clare[1], Andrew Cassidy[1], Andrew Steven[1], Kelly L. Johnston[1], John McCall[2], Louise Ford[1], Stephen A. Ward[1] & Mark J. Taylor[1‡]

[1]Research Centre for Drug and Diagnostics, Department of Parasitology, Liverpool School of Tropical Medicine, Pembroke Place, Liverpool L3 5QA, UK.

[2]TRS Laboratories, Athens, Georgia

*equal contributorship

Correspondence and requests for materials should be addressed to J.H. (email: Janet.Hemingway@lstmed.ac.uk)

Table S1 – *B. malayi* adult parasites loads post-treatment with MIN or ABZ monotherapies in CB.17 SCID mice

Variation in *B. malayi* worm burden between control and drug treatment groups was not significantly different when analyzed by Kruskal-Wallis 1way ANOVA (Kruskal Wallis statistic 5.81, total worm burden; 4.49, female worm burden; 4.78, male worm burden))

group	duration	Group n	Median total worm burden<br>(sum, min-max)	Median female worm burden<br>(sum, min-max)	Median male worm burden<br>(sum, min-max)
Vehicle	7d	4	11<br>(46, 3-21)	4<br>(19, 3-8)	6.5<br>(23, 1-9)
MIN 25MK*bid*	7d	4	13.5<br>(57, 8-22)	4.5<br>(23, 2-12)	6.5<br>(25, 4-8)
ABZ 5MK*bid*	7d	4	9<br>(37, 6-13)	3<br>(12, 0-6)	5<br>(18, 1-7)
ABZ 10MK*bid*	7d	4	5<br>(25, 3-12)	2<br>(12, 2-6)	2<br>(9, 1-4)
ABZ 20MK*bid*	7d	4	14.5<br>(55, 7-19)	6<br>(26, 3-11)	3.5<br>(19, 2-10)

Table S2 – *B. malayi* adult parasite loads 8 months post-treatment with ABZ, MIN or MIN+ABZ combination in gerbils

Variation in *B. malayi* worm burden between control and drug treatment groups was not significantly different when analysed by Kruskal-Wallis 1way ANOVA (Kruskal Wallis statistic = 0.6804, total worm burden; 2.41, female worm burden; 1.272, male worm burden)

group	duration	Group n	Median worm burden<br>(sum, min-max)	Median female worm burden<br>(sum, min-max)	Median male worm burden<br>(sum, min-max)	Mean mf load<br>(SEM)
Vehicle	15d	6	7.5<br>(45, 1-13)	4.5<br>(23, 0-6)	3<br>(22, 1-7)	1.925×10^6<br>0.487×10^6
ABZ 13MK*qd*	15d	6	11<br>(52, 4-16)	7<br>(30, 2-9)	4<br>(22, 2-8)	1.385×10^6<br>0.172×10^6
MIN 100MK*qd*	15d	6	9.5<br>(58, 7-12)	5.5<br>(33, 5-6)	4.5<br>(25, 1-7)	1.1×10^6<br>0.237×10^6
MIN 100MK*qd* +<br>ABZ 13MK*qd*	15d	6	9<br>(46, 2-12)	5<br>(29, 2-8)	3.5<br>(17, 0-5)	0.426×10^6<br>0.200×10^6

Observed Vs. Predicted Plot for final ABZ-SOX model

From figure S1 it can be seen that a one compartment model provides a good fit for the ABZ-SOX rich sampling data from both monotherapy and combination drug regimens.

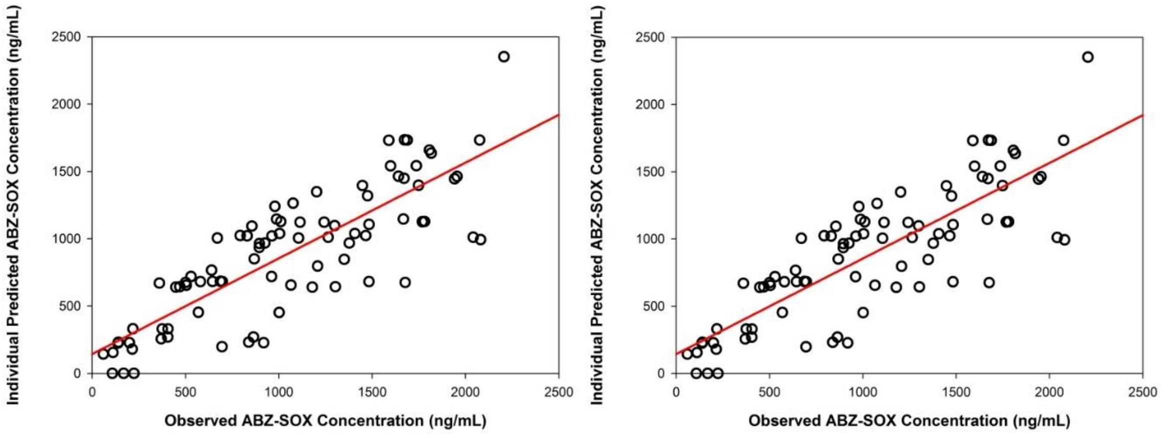

Figure S1 Individual observed vs. predicted values for **a)** 5 day 5mg/Kg *bid* ABZ monotherapy (R^2=0.68) and b) 5 mg/Kg bid combination therapy dose (R^2=0.67).

Simultaneous pharmacokinetic modelling of ABZ-SOX and ABZ-SON metabolites.

Pharmacokinetic models simultaneously fitting the drug concentrations of the pharmacologically active primary metabolite, ABZ-SOX and the inactive secondary metabolite, ABZ-SON were constructed for the 5 day 5 mg/Kg *bid* PK datasets for monotherapy and combination therapy regimen. The final model incorporated an absorptive gut compartment, central compartment and a peripheral compartment, with elimination on both the central and peripheral compartments as shown in equations 1a, 1b and 1c, respectively:

$$\frac{dX_1}{dt} = -k_a X_1 \qquad (1a)$$

$$\frac{dX_2}{dt} = k_a X_1 - \left(\frac{CL_1}{V_2}\right) X_2 - k_{cp} X_2 \qquad (1b)$$

$$\frac{dX_3}{dt} = k_a X_1 - \left(\frac{CL_2}{V_2}\right) X_3 \qquad (1c)$$

Where, X_1, X_2 and X_3 are amounts of ABZ, ABZ-SOX and ABZ-SON in the absorptive, central and peripheral compartments, respectively. The parameters k_a, k_{cp}, CL_1, V_1, CL_2, V_2 denote the lumped absorption rate constant, the rate constant for conversion from ABZ-SOX to ABZ-SON and apparent clearance and volume of distribution parameter on the central and peripheral compartments, respectively.

Table S3 shows the dose specific pharmacokinetic parameters for the two compartment models simultaneously fitting ABZ-SOX and ABZ-SON. It can be seen that the PK parameters

for ABZ administered at 5 mg/Kg *bid* for 5 days as a monotherapy or in combination with a bioequivalent regimen of rifampicin, are not statistically significantly different. It can be seen from figure S2 and S3 that the richly sampled drug concentrations are predicted accurately.

Table S3. Pharmacokinetic parameters ABZ monotherapy and combination therapy treatments, parameters calculated by simultaneously modelling ABZ-SOX and ABZ-SON administered ABZ at 5 mg/Kg .

PK Parameter	5 mg/Kg *bid* monotherapy Mean ± S.D. (*n=8*)	5 mg/Kg *bid* combination therapy Mean ± S.D. (*n=5*)
CL_1/F (*mL/hr/kg*)	269.1±235.7	293.0±158.4
V_1/F (m*L/kg*)	2696.0±923.1	2953.8±684.3
CL_2/F (*mL/hr/kg*)	2771.9±818.0	1791.6±395.7
V_2/F (m*L/kg*)	1593.7±796.6	553.7±173.0

The visual predictive check (Figure S2) for both monotherapy (a,b) and combination (c,d) models shows most data points lying with the 95% confidence intervals of the simulated profiles. Furthermore, linear regression of the observed Vs. predicted concentration plots for the 2-compartment models described above (Figure S3) show a good fit for both the primary and secondary metabolites concentrations, as indicated by the R-squared value. These models were not used further as the model fitting the pharmacologically active primary metabolite only was a slightly better fit and parameters were simpler to interpret.

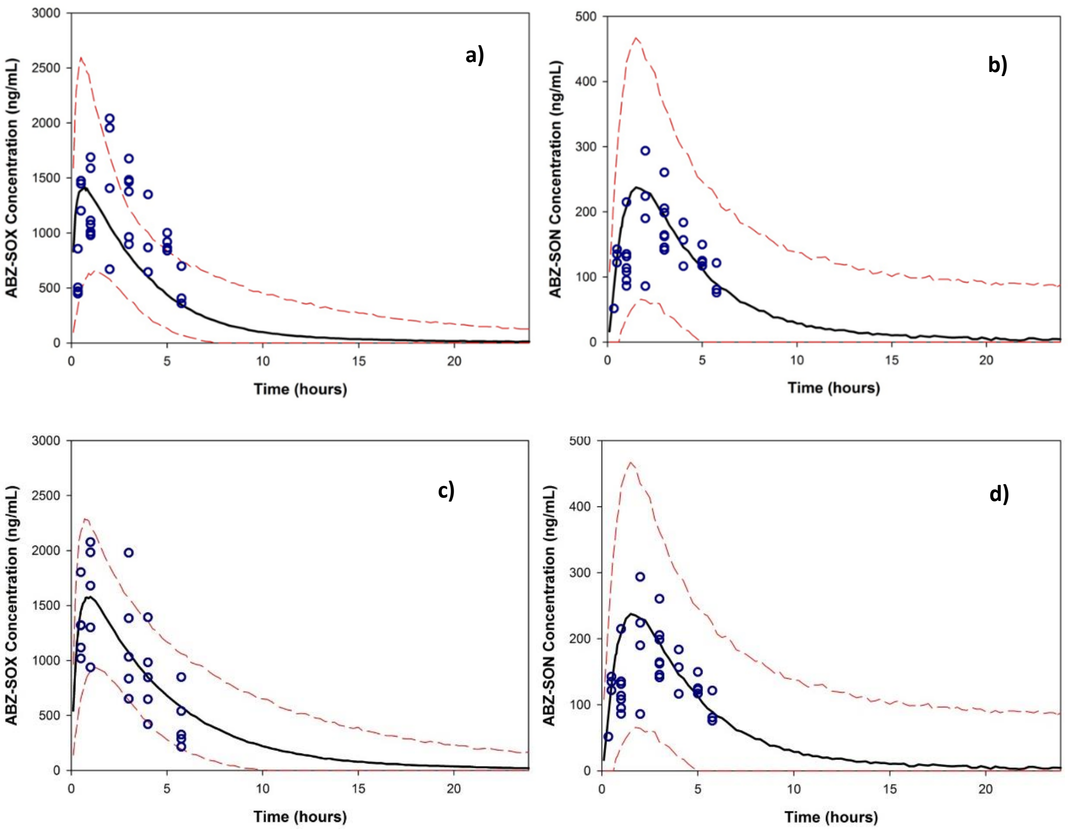

Figure S2 Simulated PK profile superposed with experimentally determined metabolite concentrations for 5 day 5mg/Kg *bid* ABZ monotherapy, **a)** albendazole sulphoxide (ABZ-SOX) concentrations **b)** albendazole sulphone

(ABZ-SON) concentrations. Also shown, simulated PK profile for 5 mg/Kg *bid* ABZ given in combination with 5 mg/Kg *qd* RIF, **c)** albendazole sulphoxide (ABZ-SOX) concentrations **d)** albendazole sulphone (ABZ-SON). For the simulated PK profiles the solid line represents the median profile and the red dashed lines represent the 5th and 95th percentile PK profiles. Experimentally determined discreet sampling concentrations are shown are dark blue circles.

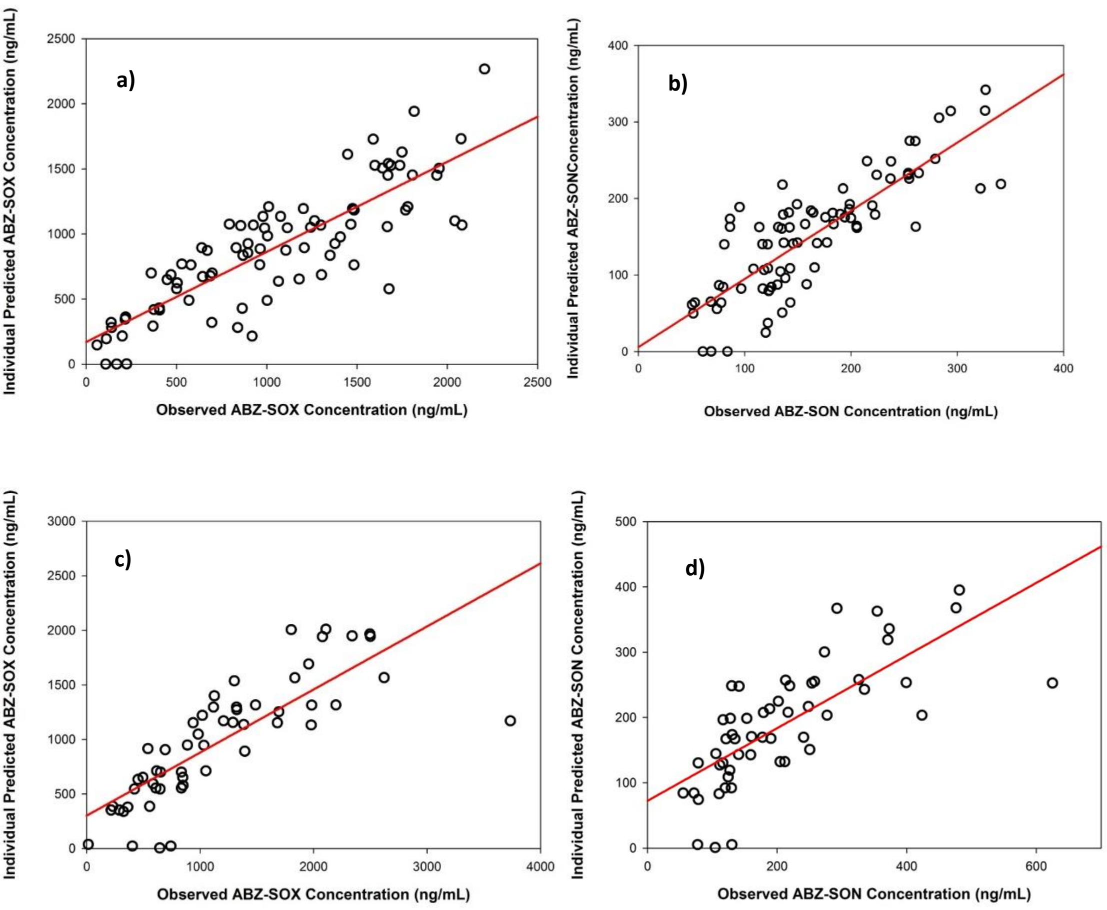

Figure S3 Individual observed vs. predicted concentration plots for 5 day 5mg/Kg *bid* ABZ monotherapy, **a)** albendazole sulphoxide (ABZ-SOX) concentrations **b)** albendazole sulphone (ABZ-SON) concentrations. Also shown, simulated PK profile for 5 mg/Kg *bid* ABZ given in combination with 5 mg/Kg *qd* RIF, **c)** albendazole sulphoxide (ABZ-SOX) concentrations **d)** albendazole sulphone (ABZ-SON).

Pharmacokinetic interactions of rifampicin+albendazole combination therapy (confirmation from sparse sampling of efficacy screens)

The drug concentrations from sparse sampling of ABZ monotherapies and ABZ+RIF combination therapies efficacy studies in the *Brugia malayi* infection model were examined to check that concentrations were within expected bounds as defined by the PK models. Figure S4 plots sparsely sampled ABZ-SOX, ABZ-SON and RIF concentrations from various efficacy studies incorporating various drug regimens against the PK profile for monotherapy and combination therapy ABZ and RIF in SCID mice. It can be seen that the drug concentration for both monotherapies and combination therapies are predominantly within 5th and 95th percentiles indicating that pharmacokinetics in the uninfected SCID mice are very indicative of those in the *Brugia malayi* infection model. When concentrations were above the 95th

percentile it can be seen that there is no systematic difference between monotherapy and combination therapy sampling points.

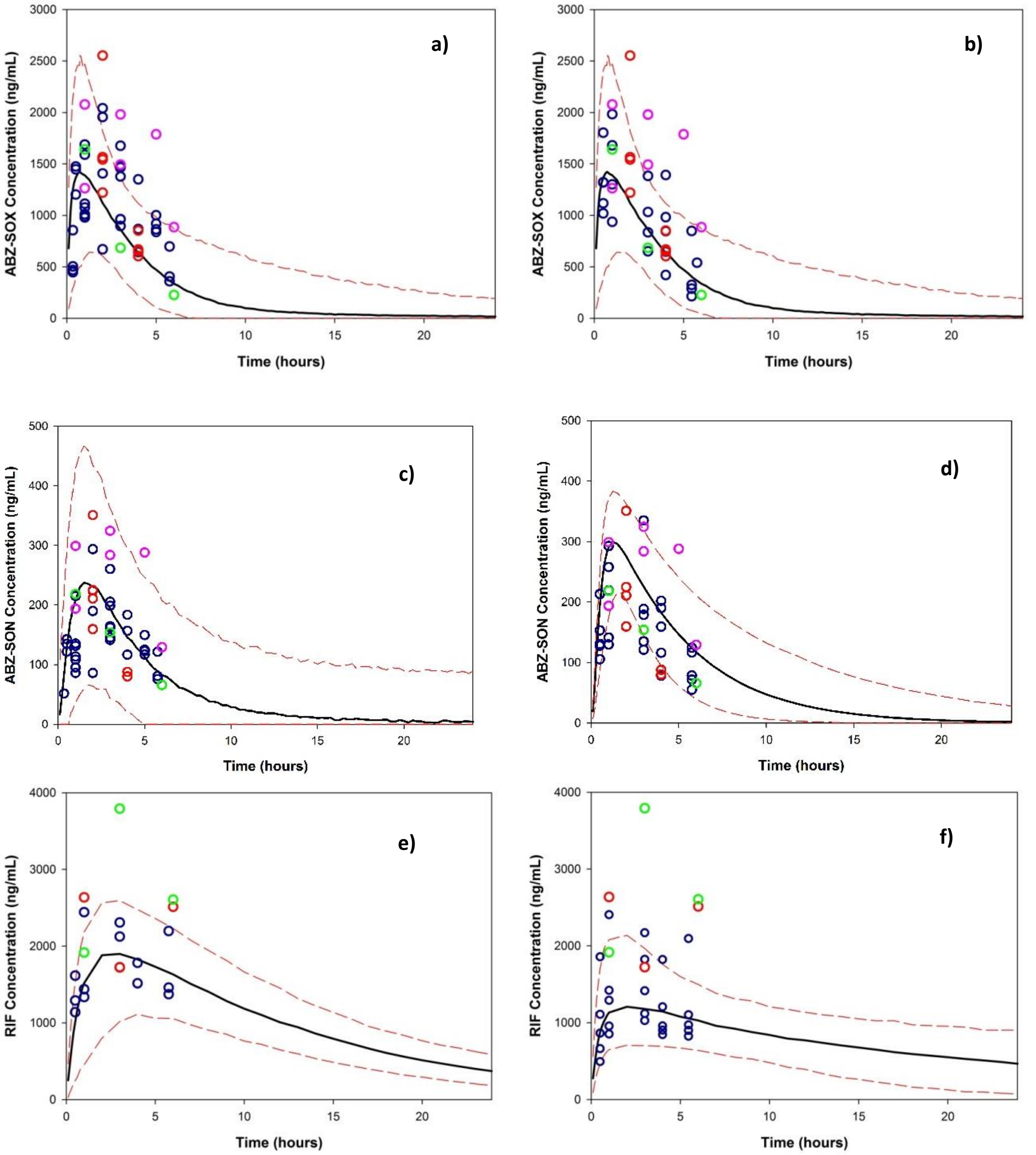

Figure S4 Simulated PK profile for (a) ABZ-SOX; 5 mg/Kg *bid* ABZ monotherapy and (b) ABZ-SOX; 5 mg/Kg *bid* ABZ given in combination with 5 mg/Kg *qd* RIF, (c) ABZ-SON; 5 mg/Kg *bid* ABZ monotherapy and (d) ABZ-SON; 5 mg/Kg *bid* ABZ given in combination with 5 mg/Kg *qd* RIF. Simulated PK profile for (d) 5 mg/Kg *qd* RIF monotherapy and (e) 5 mg/Kg *qd* RIF given in combination with 5 mg/Kg *bid* ABZ. Simulated PK profiles are superposed with sparsely sampled ABZ-SOX or RIF concentrations from *Brugia malayi* efficacy screens. The lines represent the median (solid black) and 5%, 95% percentiles (dashed red) of the PK prediction based on each PK model. Dark blue open circles represent rich sampled data used to build each model. Red, green and purple circles represent sparsely sampled ABZ-SOX or RIF concentrations from 5 mg/Kg bid ABZ monotherapy, 5 mg/Kg *bid* ABZ+5 mg/Kg *qd* RIF combination therapy and 5 mg/Kg *bid* ABZ+35 mg/Kg *qd* RIF combination therapy.
